# Guided implantation of a leadless left ventricular endocardial electrode and acoustic transmitter using computed tomography anatomy, dynamic perfusion and mechanics, and predicted activation pattern

**DOI:** 10.1016/j.hrthm.2023.07.007

**Published:** 2023-11

**Authors:** Baldeep S. Sidhu, Angela W.C. Lee, Justin Gould, Bradley Porter, Benjamin Sieniewicz, Mark K. Elliott, Vishal S. Mehta, Nadeev Wijesuriya, Abdoul A. Amadou, Gernot Plank, Ulrike Haberland, Ronak Rajani, Christopher A. Rinaldi, Steven A. Niederer

**Affiliations:** ∗ School of Biomedical Engineering and Imaging Sciences, King’s College London, London, United Kingdom; † Guy’s and St Thomas’ NHS Foundation Trust, London, United Kingdom; ‡ Siemens Healthcare GmbH, Forchheim, Germany; § Medical University of Graz, Graz, Austria; ‖ National Heart and Lung Institute, Imperial College London, Hammersmith Hospital, London, United Kingdom; ¶ The Alan Turing Institute, London, United Kingdom

**Keywords:** Cardiac resynchronization therapy, Endocardial pacing, Guiding endocardial pacing, Hemodynamic assessment, WiSE-CRT

## Abstract

**Background:**

The WiSE-CRT System (EBR systems, Sunnyvale, CA) permits leadless left ventricular pacing. Currently, no intraprocedural guidance is used to target optimal electrode placement while simultaneously guiding acoustic transmitter placement in close proximity to the electrode to ensure adequate power delivery.

**Objective:**

The purpose of this study was to assess the use of computed tomography (CT) anatomy, dynamic perfusion and mechanics, and predicted activation pattern to identify both the optimal electrode and transmitter locations.

**Methods:**

A novel CT protocol was developed using preprocedural imaging and simulation to identify target segments (TSs) for electrode implantation, with late electrical and mechanical activation, with ≥5 mm wall thickness without perfusion defects. Modeling of the acoustic intensity from different transmitter implantation sites to the TSs was used to identify the optimal transmitter location. During implantation, TSs were overlaid on fluoroscopy to guide optimal electrode location that were evaluated by acute hemodynamic response (AHR) by measuring the maximal rate of left ventricular pressure rise with biventricular pacing.

**Results:**

Ten patients underwent the implantation procedure. The transmitter could be implanted within the recommended site on the basis of preprocedural analysis in all patients. CT identified a mean of 4.8 ± 3.5 segments per patient with wall thickness < 5 mm. During electrode implantation, biventricular pacing within TSs resulted in a significant improvement in AHR vs non-TSs (25.5% ± 8.8% vs 12.9% ± 8.6%; *P* < .001). Pacing in CT-identified scar resulted in either failure to capture or minimal AHR improvement. The electrode was targeted to the TSs in all patients and was implanted in the TSs in 80%.

**Conclusion:**

Preprocedural imaging and modeling data with intraprocedural guidance can successfully guide WiSE-CRT electrode and transmitter implantation to allow optimal AHR and adequate power delivery.

## Graphical abstract

**Figure:**
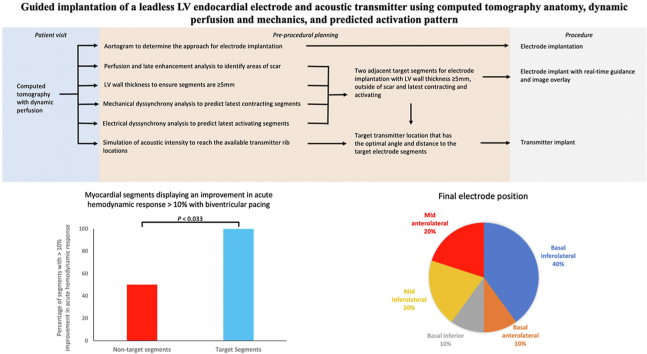

## Introduction

Cardiac resynchronization therapy (CRT) improves symptoms and reduces mortality, but even in carefully selected cases, ∼30% fail to improve. Endocardial pacing within the left ventricle can access faster endocardial conduction, enables the latest mechanically and electrically activating segments to be targeted, and can avoid myocardial scar.^1^ The WiSE-CRT System (EBR Systems, Inc, Sunnyvale, CA) allows leadless endocardial left ventricular (LV) pacing to achieve CRT.^2^ It consists of 3 separate components: the ultrasound transmitter connected to a battery and a separate endocardial electrode. Patients must have a coimplant in situ capable of continuous right ventricular (RV) pacing. The transmitter must be implanted with a shallow angle to the electrode and be in close proximity to ensure reliable biventricular pacing and improved battery longevity. The location of the electrode is an important factor for determining patient response. For effective planning, the location of both the electrode and the transmitter must be determined from preprocedural imaging.

Preprocedural cardiac magnetic resonance (CMR) imaging targeting late activating segments and avoiding scar has been shown to successfully predict the optimal sites for electrode implantation^3^; however, guiding implants with CMR imaging is limited because of artifact from the coimplant. Retrospective gated cardiac tomography can be used to identify late activating mechanical regions for targeting electrode location, but this does not identify scar regions.^4^ Cardiac computed tomography with dynamic perfusion (CCTP) with short acquisition times and excellent temporal and spatial resolution may allow guidance without the constraints of CMR imaging.

The transmitter is always implanted first, and therefore once implantation has occurred, this may limit the potential endocardial sites available.^5^ Prior studies have shown that an increased separation between the electrode and the transmitter results in failure of biventricular pacing.^2^ Once the target location of the electrode is known, preprocedural calculation of the acoustic intensity at the target electrode site for each potential transmitter site allows the optimal transmitter site to be selected, which may result in reliable right ventricualr (RV) tracking, biventricular pacing, and improved outcomes.

The WiSE-CRT procedure is associated with significant complications including cardiac tamponade in 2.2% and arterial access complications in 4.4%.^2^ Reducing the risk of these complications is critical. The electrode should be implanted in a segment with an LV wall thickness of ≥5 mm to avoid the risk of LV perforation. This can be hard to assess across the LV wall with echocardiography. Furthermore, assessment of arterial dimensions may help to reduce vascular complications.

The aim of this study was to determine whether CCTP-derived anatomy, dynamic perfusion and mechanics, and predicted activation pattern could identify the optimal sites for both transmitter and electrode implantation and allow real-time guidance for electrode implantation. We used CCTP to predict the optimal endocardial pacing sites by avoiding poorly perfused regions with an LV wall thickness of <5 mm while targeting measured mechanical and predicted electrical late activating segments. CCTP was also used to allow the acoustic intensity to be calculated, enabling the optimal transmitter site to be selected that would result in reliable RV tracking and biventricular pacing for the targeted electrode segment. We performed a prospective acute hemodynamic response (AHR) study in patients undergoing WiSE-CRT implantation with CCTP real-time guidance with image overlay for electrode implantation.

## Methods

### Study design

The study received ethical approval (18/LO/0752) and was conducted in accordance with the Declaration of Helsinki. Inclusion criteria included patients 18 years and older, indication for CRT,^6^ coimplant capable of RV pacing, and indication for WiSE-CRT implantation according to whether they were previously untreatable, a high-risk upgrade, or a CRT nonresponder. *CRT nonresponders* were defined as patients who had no change or worsening of symptoms after 6 months of CRT and whose absolute LV ejection fraction improved by <5%. Exclusion criteria included an estimated glomerular fraction rate of <30 mL/(min·1.73 m^2^), severe asthma or chronic obstructive pulmonary disease, significant aortic valve disease, and insufficient intercostal space (ICS) for transmitter implantation.

### Acoustic window screening

Acceptable transmitter intercostal implant locations were identified using ultrasound. Candidate locations were evaluated for an adequate window that had no lung encroachment during maximal inspiration and a shallow angle to the posterolateral wall of the left ventricle. An angle between the probe and the basal posterolateral wall of <30°, a distance of <12 cm, and an LV wall thickness of ≥5 mm were acceptable.

### Analysis of perfusion images

**Figure 1:**
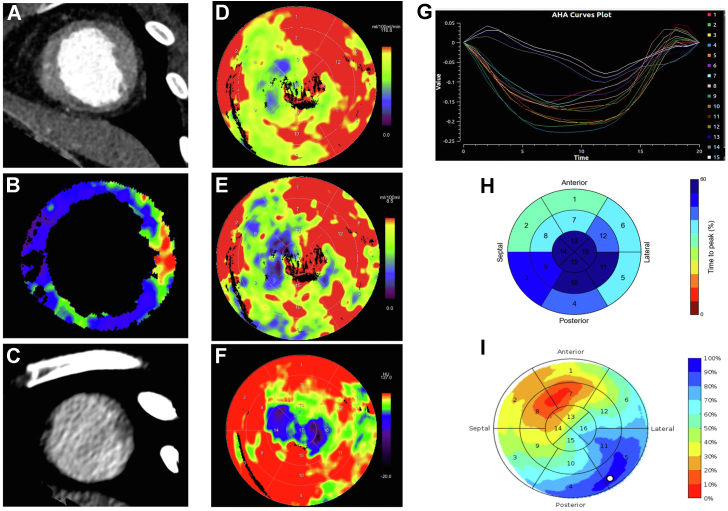
Target segments for electrode implantation. Thinning of the apical anterior and inferior segments are in shown in (**A**), with the corresponding reduced myocardial blood flow in (**B**) and late iodine enhancement in (**C**). Myocardial perfusion (**D:** myocardial blood flow; **E:** perfused capillary blood volume; **F:** average enhancement) showed significant hypoperfusion in the basal to apical anteroseptal, anterior, and inferior segments. Mechanical activation is shown in (**G**) and (**H**), with the latest time to peak contraction in the mid inferior and inferolateral segments. Panel (**I**) shows that the latest electrical activating segment was between the basal to mid inferolateral segments. Combining this information together predicted the target segments of the basal to mid inferolateral segments. The left ventricular wall thickness was ≥0.5 cm.

All CCTP scans were performed preprocedurally using the SOMATOM Force dual source scanner (Siemens Healthineers, Germany) with protocol listed in the Online Supplement. Perfusion was analyzed using a dedicated software (CT Myocardial Perfusion, Siemens Healthineers), and a motion correction algorithm was applied. The LV myocardium was segmented automatically using anatomical landmarks, Hounsfield unit–based thresholding combined with peak enhancement analysis.^7^ The arterial input function was sampled from the descending aorta in both cranial and caudal sections, and time-attenuation curves were created for each myocardial voxel within the volume of interest. A compartmental model of intravascular and extravascular space was created by applying a dedicated parametric deconvolution technique to the time-attenuation curves, enabling calculation of myocardial blood flow, perfused capillary blood volume, and average enhancement^7^^,^^8^ (Figure 1). Areas with significant hypoperfusion were considered scarred, as all patients had significant coronary disease excluded before enrollment. Delayed phase scans were analyzed to identify areas of late enhancement.

### Identifying latest mechanical and electrical action times and wall thickness

Mechanical dyssynchrony analysis was performed on Cardiac Electro-Mechanics Research Group Application (CemrgApp; www.cemrgapp.com).^9^, ^10^, ^11^, ^12^, ^13^ Using cardiac computed tomography (CT) angiograms, an image registration warping field was applied to a triangulated LV endocardial mesh. Semiautomated LV cavity segmentation was performed and used to track the LV endocardial longitudinal and circumferential strain and local area change throughout the cardiac cycle (Figure 1). Segments were excluded if their volume curves were flat and the latest mechanically activating segment was the latest time to peak contraction. Electrical activation was calculated using a similar protocol to previously reported.^14^ Patient-specific models were created using the AxSeg v4.19 tool (Siemens Healthineers), and conduction patterns were simulated by pacing from the RV lead. The longest electrical delay was taken as the latest electrically activating segment (Figure 1). LV wall thickness was calculated from the CT-derived patient-specific model and regions with wall thickness < 5 mm labeled as thin.

### Targeting sites for electrode and transmitter implantation

**Figure 2:**
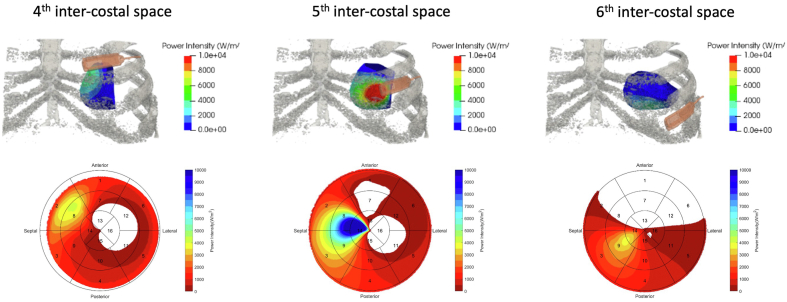
Acoustic intensity. The acoustic intensity of the transmitter when placed onto the viable intercostal space (ICS) was modeled. A transmitter was placed onto a 3-dimensional reconstruction of the rib cage, and the corresponding acoustic intensity was shown on a 16-segment bull’s-eye plot. This patient had 3 available ICSs. *White areas* had no power coverage, and *blue areas* had the greatest acoustic intensity likely to yield reliable biventricular pacing. If the target segment was basal anterolateral, then the sixth ICS should not be used, as this would not provide biventricular pacing.

**Figure 3:**
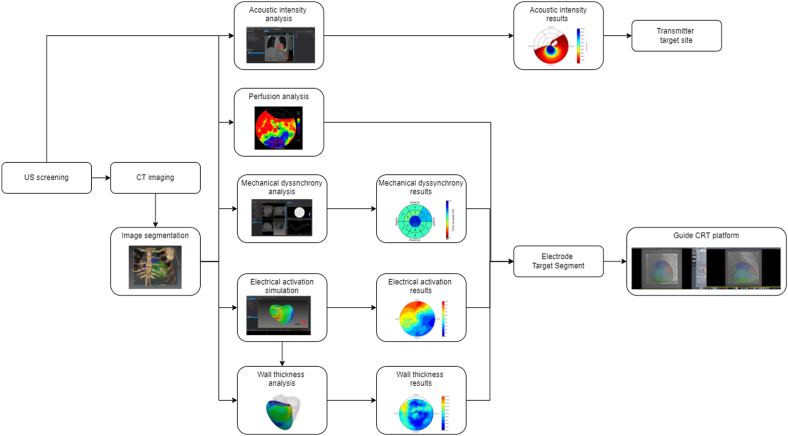
Flowchart of the planning protocol. CRT = cardiac resynchronization therapy; CT = computed tomography; US = ultrasound.

Thickness, perfusion, and activation times were defined on American Heart Association regions or segments. The latest mechanically and electrically activating segments outside the thin or myocardial scar segments were chosen as target sites for endocardial electrode implantation. On the basis of this analysis, 2 adjacent myocardial segments were chosen. Using a 3-dimensional reconstruction of the rib cage and left ventricle, the transmitter was virtually placed onto a suitable ICS and the acoustic intensity at the target segments calculated (Figure 2). The acoustic intensity of the transmitter was calculated on the basis of the depth and distance between the target segments and the transmitter. The optimal site for transmitter implantation that yielded the greatest predicted acoustic intensity was selected, and in patients who had only 1 available ICS for implantation, the acoustic intensity was calculated to ensure adequate coverage of the target segments (Figure 3).

### Procedure and hemodynamic assessment

The transmitter was implanted at a suitable ICS on the basis of acoustic window screening and the battery in the adjacent mid-axillary line. After this, the electrode was implanted via a retrograde aortic or transseptal approach as previously described (Online Supplement).^15^ The Guide CRT platform (Prototype, Siemens Healthineers) was used to overlay the electrode target segments on live fluoroscopy and has previously been shown to provide accurate image overlay for device implantation^9^^,^^10^^,^^16^, ^17^, ^18^ (Online Supplement). Invasive hemodynamic recordings were made by placing 0.014-in high-fidelity wireless PressureWire X (Abbott) inside the left ventricle and using CoroFlow (Coroventis, Sweden). A 6-F roving decapolar catheter (Abbott) provided biventricular pacing using a similar protocol to previously reported.^19^^,^^20^ Biventricular pacing was produced by timing LV pacing via the decapolar catheter with RV pacing via the coimplant. Biventricular capture was confirmed by a change in QRS morphology and duration compared with the QRS morphology of intrinsic rhythm, RV and LV pacing. For AHR measurements, baseline pre– and post–biventricular pacing was performed for a minimum of 10 seconds with additional beats recorded for ectopy and the mean change in the maximal rate of LV pressure rise (dP/dt_max_) was taken as the reference. *AHR* was defined as the percent change from the reference dP/dt_max_ to the mean biventricular dP/dt_max_. AHR was assessed in different endocardial segments to determine the site of greatest improvement in dP/dt_max_, including the target segments, but also areas of suspected myocardial scar. After these recordings, the decapolar catheter was removed and the electrode placed in the target segment with the greatest improvement in dP/dt_max_. The outcome of this study was the evaluation of acute CRT response, which was assessed using AHR (dP/dt_max_).

### Statistical analysis

Discrete data are presented as count (percentage) and continuous data as mean ± SD for normally distributed variables and as median (interquartile range) for non-normally distributed variables. When investigating the change from baseline variables, the paired sample *t* test was used for normally distributed data and the Wilcoxon signed-rank test for non-normally distributed data. The χ^2^ test was used for among-group comparisons, or if the expected cell count was <5, then the Fisher exact test was used. A 2-sided *P* value of <.05 was considered statistically significant. Statistical analyses were performed using Prism version 8 (GraphPad Software Inc., CA) and SPSS version 25 (IBM Switzerland, Switzerland).

### Results

Eleven patients were recruited, and baseline demographic characteristics are provided in Table 1. Indications included the following: 55% previously untreatable, 18% high-risk upgrade, and 27% CRT nonresponders. The mean age was 65 ± 7 years; 8 (73%) were male; and 6 (55%) had ischemic heart disease. All patients underwent CCTP, and 1 patient withdrew before the implantation procedure. All 10 remaining patients successfully underwent WiSE-CRT implantation, with biventricular pacing confirmed in all patients. One patient had a periprocedural cerebrovascular accident.

**Table 1:** Baseline patient demographic characteristics (N =11)

Characteristic	Value
Age (y)	65 ± 7
Male sex	8/11 (73)
Ischemic cardiomyopathy	6/11 (55)
Comorbidities	
Coronary artery bypass grafting	2/11 (18)
Atrial fibrillation	5/11 (45)
Hypertension	4/11 (36)
Diabetes mellitus	5/11 (45)
Chronic kidney disease	2/11 (18)
Indication	
Previously untreatable	6/11 (55)
High-risk upgrade	2/11 (18)
CRT nonresponder	3/11 (27)
New York Heart Association functional class	2.9 ± 0.3
Minnesota Living With Heart Failure Questionnaire score	61 ± 23
Sinus rhythm	6/11 (55)
QRS morphology	
Left bundle branch block	4/11 (36)
Biventricular pacing	3/11 (27)
RV pacing	4/11 (36)
QRS duration (ms)	170 ± 28
Echocardiography	
LV ejection fraction (%)	25 ± 7
LV end-systolic volume (mL)	153 ± 68
LV end-diastolic volume (mL)	202 ± 7
6-minute walk distance (m)	295 ± 143
Medications	
Angiotensin-converting enzyme inhibitor/angiotensin receptor blockers or angiotensin receptor-neprilysin inhibitor	11/11 (100)
β-Blockers	10/11 (91)
Mineralocorticoid receptor antagonist	10/11 (91)
Anticoagulation	6/11 (55)

Values are presented as mean ± SD or n (%).

CRT = cardiac resynchronization therapy; LV = left ventricular; RV = right ventricular.

### CCTP

All patients tolerated the detailed imaging protocol, and images were analyzed to produce targets sites for the transmitter and electrode. In 1 patient, the equipment used to administer adenosine malfunctioned and stress perfusion was not undertaken. The mean radiation dose-length product was 1510 mGy·cm (1142–1717 mGy·cm); the scanning time was 64 seconds; and patients were on the table for 15 minutes. One patient had a dilated left ventricle, and their dimensions were outside the perfusion imaging limit of 10.5 cm. After the test scan, perfusion imaging was adjusted to ensure only the true apex was missed in assessment as pacing in this location was unlikely to yield positive outcomes. Eighty percent of patients were stressed with adenosine and 20% with regadenoson. All patients were adequately stressed before acquisition of perfusion images: all patients experienced symptoms, and 90% had increased heart rate ≥10%. No patients had any complications related to the imaging protocol. Myocardial scar was inferred in all 6 patients with ischemic cardiomyopathy (Table 2), with areas of significant hypoperfusion identified on myocardial blood flow, perfused capillary blood volume, or average enhancement. All areas of hypoperfusion corresponded with segments that were hypokinetic or akinetic and thinned. Late iodine enhancement was seen in only 2 patients. One patient had historical CMR and perfusion defects identified on CCTP-matched late gadolinium enhancement.

**Table 2:** Cardiac computed tomography with dynamic perfusion (N = 11)

Variable	Value
Baseline systolic blood pressure (mm Hg)	116 ± 13
Baseline heart rate (beats/min)	72 (66–74)
Final dose of the pharmacological stress agent	
Adenosine 140 μg/(kg·min)	5/10 (50)
Adenosine 175 μg/(kg·min)	2/10 (20)
Adenosine 210 μg/(kg·min)	1/10 (10)
Regadenoson 400 μg	2/10 (20)
Features of adequate stress	
Symptoms	10/10 (100)
Increase in heart rate ≥10%	9/10 (90)
Scar characteristics in ischemic patients	
LV wall thinning, hypokinetic, or akinetic	6/6 (100)
Perfusion abnormality	6/6 (100)
Late iodine enhancement	2/6 (33)
Left anterior descending artery territory∗	5/6 (83)
Left circumflex artery territory∗	3/6 (50)
Right coronary artery territory∗	3/6 (50)

Values are presented as mean ± SD, median (interquartile range), or n/total n (%).

LV = left ventricular.

∗ Three of 6 patients (50%) had ≥1 coronary artery territory involved.

Analysis of LV wall thickness demonstrated a substantial number of segments (mean 4.8 ± 3.5 segments per patient) were <5 mm and deemed unsuitable for electrode implantation. Aortography demonstrated significant peripheral vascular disease in 1 patient, which was previously unknown and therefore a transseptal approach was successfully undertaken.

Analysis was completed within 2 ± 1 days between the scan and implantation.

### Transmitter implantation

Preprocedural echocardiography identified 7 patients with >1 suitable ICS (64%), 3 patients with 3 suitable ICSs (27%), and 4 had only 1 suitable ICS (36%) to position the transmitter. In all cases, the transmitter was implanted within the suitable ICS that was recommended on the basis of preprocedural CT and modeling analysis of acoustic intensity at the electrode target segments.

### Real-time guidance of the electrode

**Figure 4:**
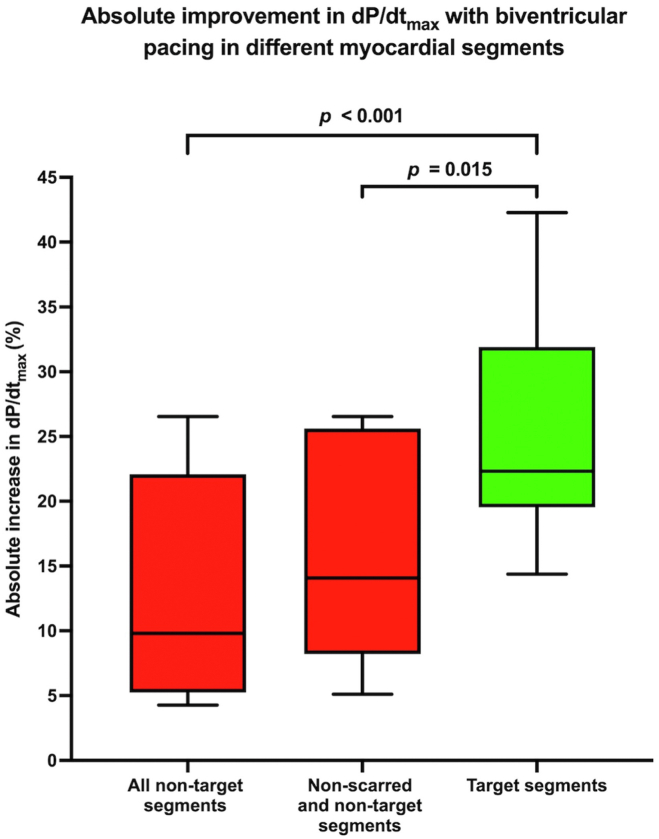
Box and whisker plot showing absolute improvement in the maximal rate of left ventricular pressure rise (dP/dt_max_) with biventricular pacing in target and nontarget segments.

A retrograde aortic approach was used in 9 patients (90%) and a transseptal approach in 1 patient (10%) because of unsuitable arterial access identified on CCTP. The latest mechanical and electrical activating sites were often similar; except in some cases, the latest electrically activating segment was in an area of predicted myocardial scar. Targeted electrode implantation including invasive hemodynamic recordings had a mean duration of 62 ± 28 minutes and a median fluoroscopic radiation dose-area product of 1058 cGy·cm^2^ (821–1820 cGy·cm^2^). Biventricular pacing within the target segments resulted in the greatest increase in AHR with a significantly greater absolute improvement in dP/dt_max_ vs nontarget segments (25.5% ± 8.8% vs 12.9% ± 8.6%; *P* < .001) (Figure 4). All target segments had an absolute improvement in AHR of >10% vs only 50% of nontarget segments (*P* = .033). Biventricular pacing within CCTP identified that poorly perfused segments resulted in either no LV capture or a minimal improvement in dP/dt_max_, and these segments always displayed the lowest improvement in AHR of all segments tested in a patient. Poorly perfused segments had an absolute improvement in dP/dt_max_ of only 5.9% and an improvement in AHR of >10% in only 6.2% of segments. Nontarget segments without poor perfusion had a significantly lower improvement in AHR than did target segments (15.4% ± 8.0% vs 25.5% ± 8.7%; *P* = .015). The final location for the endocardial electrode included 4/10 basal inferolateral, 1/10 basal anterolateral, 1/10 basal inferior, 2/10 mid inferolateral, and 2/10 mid anterolateral segments. In patients who had a functioning or nonfunctioning epicardial LV lead, the electrode was placed in a different myocardial segment in 6 of 7 patients as this yielded the greatest improvement in dP/dt_max_.

In patients who had the WiSE-CRT System implanted for CRT nonresponse, the electrode was placed in a different myocardial segment in all patients and endocardial pacing compared with epicardial pacing resulted in a nonsignificant absolute improvement in dP/dt_max_ (24.3% ± 9.2% vs 14.5% ± 18.3%; *P* = .570).

The endocardial electrode was guided to the preprocedural imaging and modeling–defined target segments, and in all cases, these segments yielded the greatest improvement in AHR. In 2 patients with nonischemic cardiomyopathy, the electrode was not deployed in the target segment because of suboptimal preanchoring pacing thresholds and insufficient electrode tenting despite multiple catheter manipulations and therefore the electrode was placed within the segment adjacent to the target. The mean QLV was 115.1 ± 29.9 ms. In all cases where the electrode was implanted in target segments, the transmitter proximity and angle were within device tolerances and all patients had evidence of RV tracking and biventricular pacing at the end of the procedure.

After 6 months of WiSE-CRT sytem pacing, 50% had a reduction in LV end-systolic volume of ≥15% and 70% improved their clinical composite score consisting of no heart failure hospitalizations, alive, and improvement in their global assessment or New York Heart Association functional class.

## Discussion

This is the first study to comprehensively perform imaging and modeling–guided leadless LV endocardial pacing with CCTP with real-time image overlay. This demonstrated the following:

1. CT anatomy, dynamic perfusion and mechanics, and predicted activation pattern predicted endocardial target segments with the greatest improvement in AHR in all patients.
2. CCTP reliably identified poorly perfused regions in all patients with ischemic heart disease, and these segments correlated with poor AHR.
3. The optimal transmitter site with the greatest acoustic intensity at the target segments was identified preprocedurally in all cases. This resulted in an acceptable angle and depth with reliable tracking between devices in those patients who had the electrode implanted within the target segment.
4. Advanced image and simulation analysis for patient-specific intraprocedural guidance can be performed within clinical timescales.

### Identifying myocardial scar

CMR remains the criterion standard for identifying myocardial scar, but its use is limited in patients undergoing WiSE-CRT implantation as the coimplant causes significant artifact and devices may be non–magnetic resonance imaging conditional. Additionally, the multiple breath-holds required for accurate analysis are limited in patients with severe heart failure. CCTP offers many advantages including quicker acquisition times and is an appealing option for such patients. The identification of myocardial scar by late iodine enhancement is often restricted to clinical trials and is limited by a lack of standardized protocols and heterogeneity in identifying scarred segments.^21^ Consistent with these findings, only 2 of 6 ischemic patients (33%) had late iodine enhancement. We used a novel protocol to identify perfusion abnormalities as a potential surrogate for scar that identified perfusion defects in all patients with ischemic heart disease. Furthermore, myocardial segments with perfusion defects exhibited minimal AHR, supporting the use of CCTP to identify myocardial scar.

### Guidance of the electrode and transmitter

Currently, patients eligible for the WiSE-CRT System must have had a previous attempt at CRT or already have a device in situ but considered high risk for CRT upgrade, for example, because of venous occlusion. Accordingly, these patients are often sicker than patients treated with conventional CRT with multiple comorbidities and ensuring that the system works reliably while targeting the optimal endocardial location is essential. The ability of endocardial pacing to target specific sites is a major advantage over epicardial pacing. Guiding pacing can improve outcomes by selecting the latest activating segments while avoiding scar,^9^^,^^10^ enabling patient-specific pacing, and in the present study, target segments displayed the greatest improvement in dP/dt_max_.

Currently, no preprocedural information is used to guide the optimal transmitter implantation site and current workflows provide no estimation of whether the acoustic intensity will result in reliable tracking. Insufficient acoustic intensity due to poor transmitter coverage has been implicated in previous device failures.^2^ The acoustic intensity is dependent on the depth and angle to the electrode; therefore, targeting the electrode to a specific site can be accomplished only after the optimal transmitter site is known and thus both must be predicted preprocedurally. This is especially true of myocardial sites other than the basal inferolateral wall, which cannot be estimated during acoustic window screening. In the present study, all patients who had their transmitter and electrode placed within the predicted implantation sites had an adequate angle and depth, resulting in reliable tracking and biventricular pacing. In 2 patients who had their electrode implanted outside the target segment in an adjacent segment, preprocedural modeling of the acoustic intensity to the final implanted segment had predicted suboptimal tracking owing to an increased device separation.

### Reducing procedure-related complications

LV endocardial pacing is associated with a significant risk of complications including cardiac tamponade and vascular complications.^2^ Although there are many causes for cardiac tamponade, an important factor is to ensure that the LV wall thickness is ≥5 mm. Acoustic window screening can provide an estimate of the basal inferolateral wall thickness, but accuracy is limited by suboptimal views and assessment of different segments is unreliable. We showed that LV wall thickness can be measured from CCTP, and this identified a substantial number of unsuitable segments. Preprocedural identification and exclusion of unsuitable segments has the potential to reduce the risk of cardiac perforation.

### Clinical perspective

This study has demonstrated that a novel computational image analysis and modeling planning protocol was able to successfully guide WiSE-CRT electrode and transmitter implantation. Target segments had the greatest improvement in AHR measured with dP/dt_max_, a metric shown to predict reverse remodeling,^20^ suggesting that guidance should result in chronic response. Preprocedural assessment of activation patterns, myocardial scar, and LV wall thickness with image integration should avoid the need for invasive hemodynamic assessment, which may reduce procedure length and complications and improve LV remodeling and patient symptoms. Assessment of the acoustic intensity of different transmitter sites ensures that the optimal sites for both devices are identified preprocedurally, and this coupled site/signal strategy has the potential to reduce procedure time and complications related to myocardial perforation while improved battery longevity reducing the need for generator changes.

### Limitations

The study size was small as patients were required to undertake multiple preprocedural and procedural assessments, potentially limiting generalizability. The percentage of RV tracked signals that actually result in an ultrasound wave causing LV stimulation cannot be fully relied on without attaching a 24-hour tape. Patients with a dilated LV may not be able to undergo perfusion imaging as their ventricles will be too large to be analyzed entirely, thus limiting CCTP in these patients, although the scan can be adjusted to capture basal segments. Analysis of CCTP requires the use of dedicated analysis programs, and this can be time- consuming. This study has shown the feasibility of image overlay with CCTP; however, larger studies will be required to assess the incremental benefits of these findings. This was predominantly an acute study and not powered to look into long-term outcomes; therefore, it is unclear what the potential magnitude of benefit would be in the clinical world should patients undergo these protocols.

## Conclusion

Real-time image overlay using a novel CT protocol, image analysis, and modeling is able to successfully guide the wireless endocardial electrode to the site of greatest improvement in AHR and the optimal transmitter location, which should allow increased device longevity. Intraprocedural guidance of the WiSE-CRT System may allow improved device longevity and patient outcomes.
