# Supplementary material for: Guided implantation of a leadless left ventricular endocardial electrode and acoustic transmitter using computed tomography anatomy, dynamic perfusion and mechanics, and predicted activation pattern
Source: Heart Rhythm. 2023 Nov;20(11):1481–8. doi: 10.1016/j.hrthm.2023.07.007 (PMC10850882; doi:10.1016/j.hrthm.2023.07.007)
Supplement: Supplementary Data [file mmc1.docx]

Supplementary File

*Protocol for computed tomography with dynamic perfusion*

All scans were performed on the Siemens SOMATOM Force dual source scanner (Siemens Healthineers, Germany). A low dose non-contrast scan was used to reduce the range of retrospective scanning. A dynamic perfusion acquisition of 30-40s (80kV, 300ref.mAs, ECG-triggered 250ms after the R-wave) was performed following a bolus of 50ml intravenous iodinated contrast (Omnipaque, GE Healthcare, USA) under pharmacological stress. Adenosine (140-210mcg/kg/min) was used to induce stress or Regadenoson (400mcg) in patients with severe asthma. Patients were considered adequately stressed if they developed symptoms or displayed an increase in heart rate ≥10% compared with baseline. Once the heart rate had normalised, another injection of iodinated contrast was given and automated descending aorta contrast-triggered ECG-gated scanning was performed with wall motion analysis in a single breath hold. Scanning parameters included a heart rate dependent pitch (0.2-0.45), gantry rotation time of 250ms tube voltage of 100 or 120 kVp, depending on the patient’s body-mass index and a tube current of 125-300mAs. A prospective high-pitched helical CT aortogram was performed to evaluate the arterial vasculature. A prospectively ECG-gated scan was acquired at 12.5 minutes from the second contrast bolus for scar analysis. The acquired cardiac CTA data was reconstructed using a medium level of advanced model based iterative reconstruction with the use a 250mm field of view, 512x512 matrix and a smooth reconstruction kernel. In the presence of significant ectopy, ECG-editing was applied.

*Real-time guidance for electrode implantation*

The Guide CRT platform (Siemens Healthineers, Germany) is a custom-build software protype and is integrated with Artis Q biplane Angiography system (Siemens Magnetom Artis Combi Suite, Siemens Healthcare GmbH, Germany) to provide real-time guidance. Data was extracted from CCTP using an automated protocol for slice registration and left ventricle segmentation. Target segments were highlighted on a three-dimensional mesh of the left ventricle and overlaid onto live fluoroscopy.

*Different approaches for electrode implantation*

The electrode can be implanted via a retrograde aortic or trans-septal approach. With a retrograde-aortic approach, access is gained via the femoral artery and the WiSE-CRT delivery equipment is passed into the left ventricle through the aortic valve. This allows access to the entire left ventricle, although positioning the electrode onto the septum can be technically challenging. With a trans-septal approach, access is gained from the femoral vein and an 8 Fr Fast-cath transseptal guiding introducer Swartz SL1 (Abbott, Minnesota) using a Baylis NRG Transseptal Needle (Baylis Medical Company, Canada) is used to gain transseptal access under transoesophageal guidance. The sheath is then upsized to a 12-Fr Transseptal Mullins introducer sheath (Medtronic, Minnesota) and is placed across the mitral valve. This allows access to the left ventricular endocardium and studies have shown the posterior and lateral walls can be accessed for electrode implantation.
